# Association of mobile device proficiency and subjective cognitive complaints with financial management ability among community-dwelling older adults: a population-based cross-sectional study

**DOI:** 10.1007/s40520-024-02697-8

**Published:** 2024-02-17

**Authors:** Suguru Shimokihara, Yuriko Ikeda, Fumiyo Matsuda, Takayuki Tabira

**Affiliations:** 1 Graduate School of Health Sciences, Kagoshima University, 8-35-1, Sakuragaoka, Kagoshima, 890-8544 Japan; 2 Research Fellowship for Young Scientists, Japan Society for the Promotion of Science, 5-3-1, Kojimachi, Chiyoda-Ku, Tokyo, 102-0083 Japan; 3 Department of Occupational Therapy, School of Health Sciences, Faculty of Medicine, Kagoshima University, 8-35-1, Sakuragaoka, Kagoshima, 890-8544 Japan; 4 Department of Physical Therapy, School of Health Sciences, Faculty of Medicine, Kagoshima University, 8-35-1, Sakuragaoka, Kagoshima, 890-8544 Japan

**Keywords:** Subjective memory complaints, Mobile device, Financial management, Community, Cross-sectional study

## Abstract

**Background:**

Mobile devices have been used by many older adults and have the potential to assist individuals with subjective cognitive complaints (SCCs) in daily living tasks. Financial management is one of the most complex daily activity for older adults, as it is easily impaired in the prodromal stage of dementia and cognitive impairment.

**Aim:**

To investigate financial management ability among older adults from SCCs and mobile device proficiency.

**Methods:**

A self-administered questionnaire was sent to 529 participants who were ≥ 65 years and regularly use mobile devices. Participants were divided into four groups based on SCC prevalence and scores of the Mobile Device Proficiency Questionnaire (MDPQ-16). Financial management abilities were compared between groups using the Process Analysis of Daily Activities for Dementia subscale. Regression model and crosstabulation table were used to investigate associations in detail.

**Results:**

A significant difference in financial management ability was observed among the four groups (*p* < 0.001), with the dual impairment group showed significantly lower than the robust and SCC groups (*p* < 0.001). Educational history, sex, and MDPQ-16 score were significantly associated with participants’ financial management ability (*p* ≤ 0.01). The proportion of participants who could use ATMs and electronic money independently was significantly lower among those with low proficiency in mobile devices (LPM), regardless of SCC (*p* < 0.05).

**Conclusion:**

The LPM group showed an impaired ability to manage their finances, particularly in situations where they would use information devices. Therefore, healthcare professionals should assess not only the SCC but also their proficiency with mobile devices to predict their impairment in activities of daily living.

**Supplementary Information:**

The online version contains supplementary material available at 10.1007/s40520-024-02697-8.

## Introduction

Older adults experience subjective cognitive complaints (SCCs) in the prodromal phase of cognitive impairment and dementia [1, 2]. SCC refers to an individual’s concerns about general memory and cognitive function, with or without cognitive impairment [3, 4]. Older adults with SCC are associated with an increased risk of developing dementia [5, 6], and adverse health outcomes such as decreased quality of life, depression, and impaired self-efficacy [7]. Moreover, one-quarter to one-half of older adults living in the community experience SCC [8]. Subjective memory impairment has been found to be associated with impairment in activities of daily living (ADL) and instrumental ADL (IADL) [9–11]. Therefore, the association with IADL impairment may be greater in SCC, which includes impairment in domains other than memory [12]. Therefore, addressing SCC can significantly impact the health and ability of older adults in conducting their IADL. Regarding IADL in older adults, financial management is impaired in patients with mild cognitive impairment and patients with mild Alzheimer’s disease [13, 14]. Moreover, even healthy older adults experience difficulties with financial management from the stage of cognitive frailty [15]. Therefore, financial management could be the most complex IADL in older adults requiring early support.

In the past, many studies have discussed the relevance of information and communication technology (ICT) and digital technology applications for the health and well-being of older adults. Telemonitoring technology has been useful in the long-term management of disease in older adults with chronic obstructive pulmonary disease [16]. The application of sensor technology in the management of falls in older adults has also been explored, with the promise of using the ICT to predict and prevent falls [17]. In addition, the enacted mobility assessment of older adults using a smartphone that tagged with the Global Positioning System is also presented [16]. Thus, the use of ICT and digital technologies has the potential to support healthy aging in older adults. Mobile devices are the most familiar and accessible interface for older adults and can provide ICT and digital technologies on a daily basis. Mobile devices are small, lightweight, and portable information terminals that can efficiently perform various tasks in daily life. Many older adults have been owning smartphones and tablets in the recent years. Approximately 61% of older adults in the United States [18] and 74.2% of those over 65 in Japan [19] own a smartphone. Mobile devices are used daily for making phone calls, exchanging messages, checking schedules, shopping, payments, and entertainment. However, older adults owning mobile devices may not use them to their full potential. Older adults use a few applications and take long time to complete tasks on mobile devices [20]. Although older adults have mobile devices, their ability to use them is limited, and their difficulties in life may vary depending on their ability to use mobile devices. However, only a few reports examine the relationship between older adults’ impairment in IADL and mobile device proficiency.

Therefore, we assessed the relationship between the financial management ability of community-dwelling older adults in terms of SCCs and their mobile device proficiency in this study. We hypothesized that older adults with higher mobile device proficiency would have lower rates of SCC and higher financial management ability. Therefore, examining this relationship would help in inferring a decline in financial management ability based on the mobile device proficiency of older adults in today’s digital society and providing appropriate social support.

## Methods

### Study design and participants

We conducted a cross-sectional study using a self-administered questionnaire. The area where this study was conducted is Kagoshima Prefecture, located in the south of Japan. We sent a self-administered questionnaire to 3000 people aged 50 or older who were members of a consumer’s co-operative (CO-OP) in Kagoshima, Japan, inviting them to participate in this study. CO-OP is a consumer organization that helps people live prosperous lives across national boundaries [21]. CO-OP Kagoshima is a community-based organization that supports various community activities such as sale of groceries and daily necessities through physical stores and home delivery. The organization is supported by investments from its members, and there were 320,000 members in 2020. In addition, 87% of CO-OP members are women and 39.2% of households in Japan are enrolled in a CO-OP [22]. The invitees could respond to this survey by filling out the questionnaire and returning it in a self-addressed envelope or scanning a QR code to respond online. The survey was conducted over a one-month period, beginning in November 2022. We received 1189 responses (39.6% response rate; 97.9% responses through mail). Among them, participants aged between 50 and 65 were more likely to be employed, use mobile devices in ADL and at work, and have a higher likelihood of achieving a ceiling score on the mobile device proficiency assessment (Figure S1). Therefore, we selected the respondents aged 65 years or older and excluded those with incomplete responses and those who did not use mobile devices.

### Operational definition of SCC

Various methods have been developed previously to assess SCC. In this study, the following five questions were used as subjective memory questions:

1. “Do you consider yourself as being forgetful?”
2. “Do you have difficulty remembering where you leave objects—like a wallet or a key?”
3. “Do you forget the names of close friends or relatives?”
4. “Do you ever forget your appointment?”
5. “Have you ever been in your neighborhood and forgotten your way?”

The four options of the five questions were scored as: Always, 2 points; Sometimes, 1 point; Rarely, 0 points; and Never, 0 points. A total score of 2 or more for the 5 items was considered to be the presence of SCC. The questionnaire is based on the Cambridge Mental Disorders of the Elderly Examination [23] and has been used partly to determine SCC in a large cohort study in Japan [6, 24, 25].

### Assessment of mobile device proficiency

We used a short version of the Mobile Device Proficiency Questionnaire (MDPQ-16) [26] to assess mobile device proficiency among participants. This assessment evaluated participants’ proficiency in eight subitems related to mobile devices, such as basics of mobile device, communication, data and file storage, internet, calendar, entertainment, privacy, troubleshooting, and software management. We defined a mobile device as a portable device that can perform many of the same tasks as a standard computer, but without the use of a physical keyboard or mouse, as reported by Roque et al. [26]. These devices include smartphones and tablet computers.

The MDPQ-16 uses a 5-point Likert scale (1 = never tried, 2 = not at all, 3 = not very easily, 4 = somewhat easily, and 5 = very easily), and the questionnaire was scored as previously reported [26, 27]. The scores ranged from 8 to 40 points; Cronbach’s α was 0.98, indicating high internal consistency for each scale. Because our mean scores of the MDPQ-16 were higher than scores in previous studies [27], we operationally defined participants with scores less than 13.5, the 25th percentile value, as having low proficiency of mobile devices (LPM). The distribution of participants’ MDPQ-16 scores in this study is shown in Figure S2.

Further, in a short survey, we asked about participants’ subjective ability to use mobile devices and the number of apps they used. We asked participants, “How well do you use your mobile device?” and participants rated their ability to use their mobile device using a 5-point Likert scale (1 = not at all, and 5 = very well). Then, participants selected the following items that apply to the applications they use on their mobile devices: messages/texting (e.g., Facebook Messenger, WhatsApp, and Line), e-mail (e.g., Gmail, Outlook), phone calls, internet browsers (e.g., Safari and Chrome), cameras, social networking services (e.g., Twitter and Instagram), video viewing (e.g., YouTube and Netflix), online games, news and weather, maps/navigation, online shopping (e.g., Amazon and Rakuten), financial transactions, content (e-books and music), auctions, electronic government communication (e.g., tax payment and address change). For selecting these apps, we referred to the items reported in the periodic censuses in Japan [19]. The subjective ability to use mobile devices and the number of applications used were used to validate the MDPQ-16 in this study.

### Assessment of financial management ability

We used the “managing finances” subscale of the Process Analysis of Daily Activity for Dementia (PADA-D) to assess participants’ ability to manage finances. The PADA-D is a reliable and valid assessment tool that can specifically determine impairment in ADL related to cognitive functions [28, 29]. The “managing finance” subscale of the PADA-D included five processes and three actions corresponding to each process. Participants checked “yes” or “no” for each subitems. The scoring method was 1 point for “yes” and 0 points for “no,” with total scores ranging from 0 to 15. The scales of the PADA-D have high internal consistency (Cronbach’s *α* = 0.96) and criterion validity. Assessing money management ability using the PADA-D allows for a detailed assessment of the actual processes that participants are able to perform in their daily lives.

### Demographical data

We obtained the demographic characteristics of the participants, such as age in years, sex, years of education, and living situation (living alone or living with others).

### Self-reported sensory impairment

We prepared a set of questions to assess subjective visual and hearing impairments. The questions were as follows: “Do you have troubles with your vision?” and “Do you have trouble hearing?” Participants who answered “yes” to each question were considered as having subjective visual or hearing impairments.

### Statistical analysis

Our participants were categorized by SCC status and mobile device proficiency as follows: robust, SCC, LPM, and dual impairment (DI). To verify the concurrent validity of the MDPQ-16, we used Pearson’s correlation between MDPQ-16 scores and subjective ability to use and the number of applications used by study participants. Due to the non-normality of the obtained scores, Kruskal–Wallis test was used to compare continuous variables, and the Steel–Dwass–Critchlow–Fligner method was used for multiple comparisons. Pearson’s Chi-square test was used for comparisons of categorical variables. A general linear regression model was created with the PADA-D financial management scores as the dependent variable to explore the factors associated with the financial management sub-item of the PADA-D. To account for the effect of sex, we also analyzed the regression model separately for males and females. In addition, we used Pearson’s Chi-squared test and residual analysis to examine the proportion of independent respondents in the financial management subitems of the PADA-D. We used the Bonferroni method to correct *p* values to account for the increased alpha error due to multiple comparisons. Statistical analysis was performed on R version 4.2.2. The significance level was set at *p* < 0.05. In the residual analysis, an absolute value of adjusted residuals greater than 1.96 was considered *p* < 0.05, and 2.56 was considered *p* < 0.01 [30].

## Results

### Characteristics of participants

**Fig. 1:**
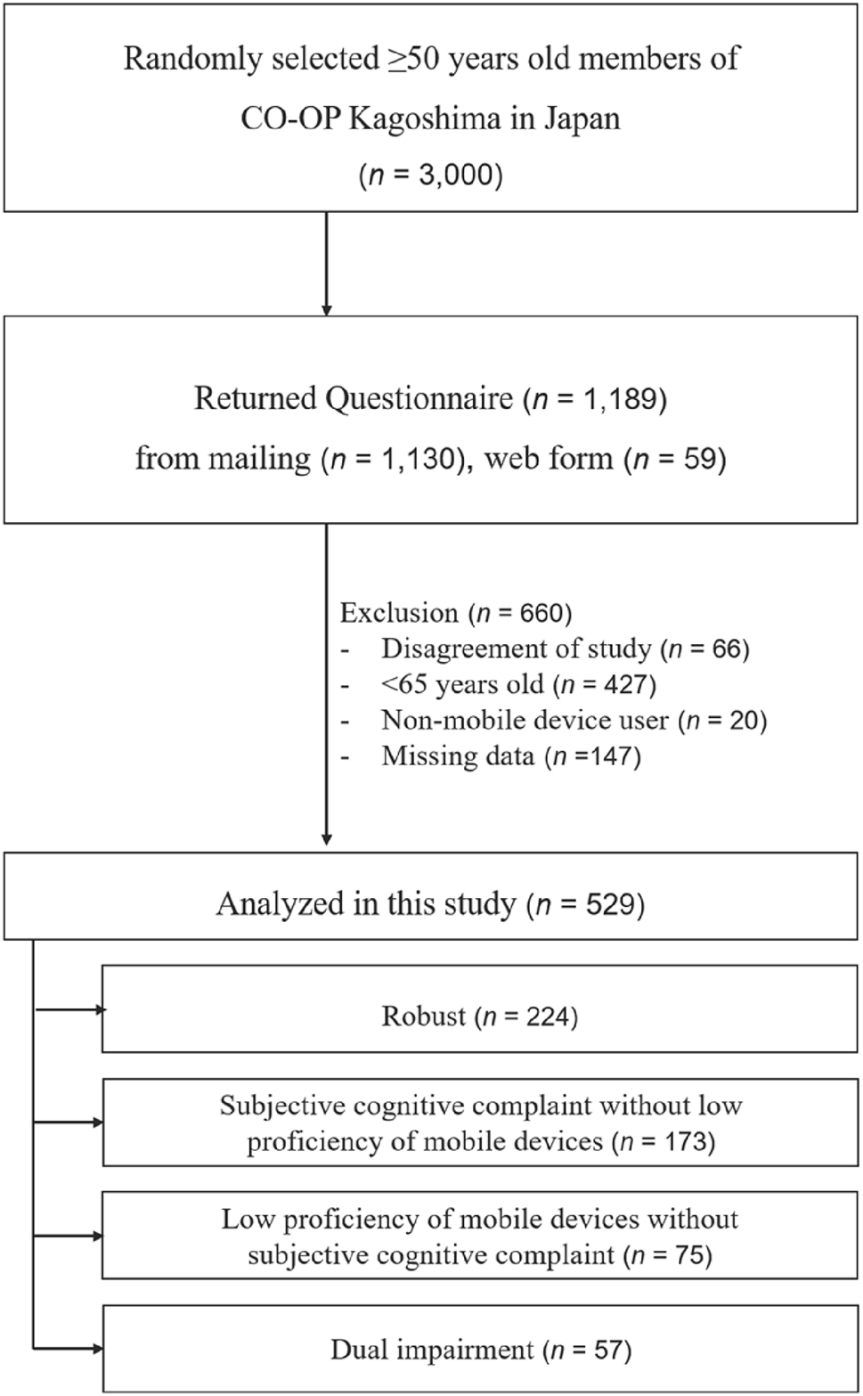
Flowchart of this study

A flow chart of this study is shown in Fig. 1. 529 participants (mean age: 72.4 ± 5.7 years; 85.1% female) were included in the analysis. Tables S1 and S2 show the characteristics of the participants with SCC or LPM. There were 230 participants with SCC (mean age 73.01 ± 6.00 years, 83% female), suggesting a higher proportion of participants with subjective sensory impairment (*p* < 0.001). There were 132 participants with LPM (mean age 75.40 ± 6.17 years, 86% female). The LPM group was significantly older (*t* = 7.34,* p* < 0.001) and had lower years of education (*p* < 0.001) than those of the non-LPM group. However, there was no difference between MDPQ-16 scores with and without SCCs and between the SCCs with and without LPM. Table 1 shows the backgrounds of the participant groups according to the presence or absence of SCC and LPM. The LPM and DI groups were significantly older and less educated than the robust and SCC groups (*p* < 0.001). The SCC group had the highest proportion of participants with subjective visual impairment (79%; *χ*^2^ = 15.7, *p* < 0.001), and the DI group had the highest proportion of participants with subjective hearing impairment (56%; *χ*^2^ = 18.5, *p* < 0.001). Significant positive correlations were found between participants’ MDPQ-16 scores and their subjective ability to use mobile devices (*r* = 0.51, *p* < 0.001) and the number of apps they used daily (*r* = 0.82, *p* < 0.001). Table S3 shows each type of mobile application used by the four groups. The most common applications used by older adults were cameras (83%), messages/texting (79%), and news and weather (77%).

**Table 1:** Characteristics of study participants

Characteristic	Overall, *n* = 529	Robust, *n* = 224^A^	SCC, *n* = 173^B^	LPM, *n* = 75^C^	DI, *n* = 57^D^	*p* value	Post-hoc
Age	72.40 (5.68)	71.12 (4.99)	71.77 (5.33)	74.37 (5.83)	76.75 (6.39)	< 0.001^a^	Robust, SCC < LPM, DI
Sex (Female)	450/529 (85%)	193 (86%)	144 (83%)	65 (87%)	48 (84%)	0.84^b^	
Education years	12.70 (2.10)	13.01 (1.86)	12.95 (2.14)	12.00 (2.42)	11.60 (1.91)	< 0.001^a^	Robust, SCC < LPM, DI
Living alone	97/529 (18%)	32 (14%)	34 (20%)	14 (19%)	17 (30%)	0.05^b^	
Subjective Visual impairment	356/529 (67%)	135 (60%)	136 (79%)	45 (60%)	40 (70%)	< 0.001^b^	
Subjective Hearing impairment	194/529 (37%)	64 (29%)	76 (44%)	22 (29%)	32 (56%)	< 0.001^b^	

Statistics presented: Mean (SD); *n*/*N* (%)

Statistical tests performed: ^a^Kruskal–Wallis test; ^b^Chi-square test of independence

Post-hoc analysis performed Dwass-Steel-Critchlow-Flinger comparisons

*SCC* subjective cognitive complain, *LPM* low proficiency of mobile devices, *DI* dual impairment

### Comparison of financial management ability

**Fig. 2:**
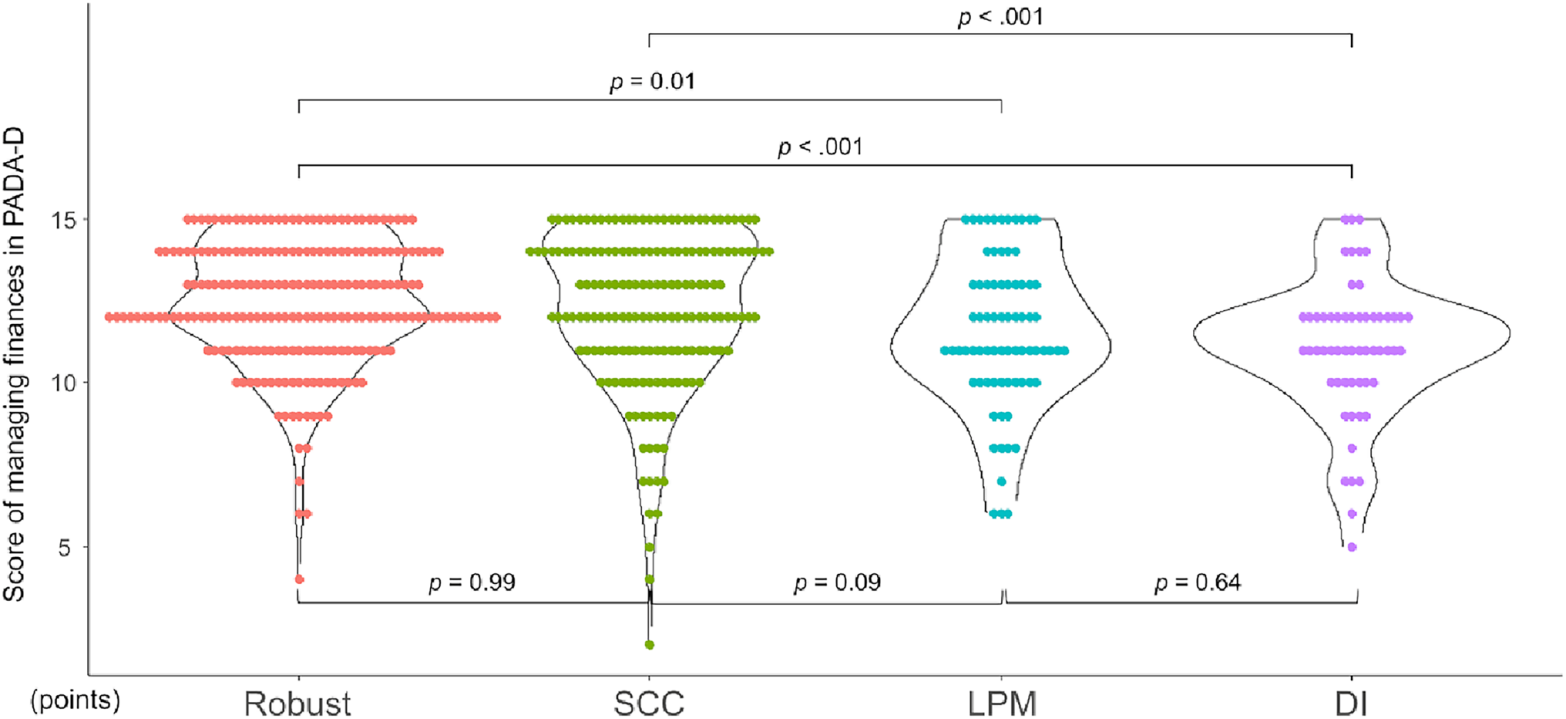
Comparison of financial management ability. Caption: Kruskal–Wallis test, and the Steel–Dwass–Critchlow–Fligner method were used comparisons. *PADA-D* process analysis of daily activity for dementia, *SCC* subjective memory complaint, *LPM* low proficiency of mobile devices, *DI* dual impairment

A comparison of the PADA-D financial management scores with and without SCC and LPM is shown in Fig. 2. There were significant differences in the PADA-D financial management scores among the four groups (χ^2^ = 25.4, *p* < 0.001, *ε*^2^ = 0.05, power = 0.91). In multiple comparisons, the DI group had significantly lower scores than the robust (*p* < 0.001) and SCC (*p* < 0.001) groups. Moreover, the robust group scored significantly higher than the LPM group (*p* = 0.001). The SCC group scored higher than the LPM group, but there was no statistically significant difference (*p* = 0.09).

### Factors associated with the ability to manage finances

Table 2 presents the results of the general linear model with the PADA-D financial management score as the dependent variable. Items that were significantly associated with the financial management score were: years of education (*β* = 0.13, *t* = 2.64, *p* = 0.01), sex (*β* = 0.38, *t* = 3.46, *p* < 0.001), and MDPQ-16 score (*β* = 0.19, *t* = 4.13, *p* < 0.001). In addition, to account for sex differences, we performed a similar analysis stratified by sex and the results remained (Table S4).

**Table 2:** Factors associated with financial management of PADA-D

Factors	*β*	95% CI		*t*	SE	*p* value
		lower	upper			
Age	− 0.003	− 0.03	0.04	0.27	0.02	0.79
Education years	0.13	0.03	0.22	2.64	0.05	0.01
Sex (ref: male)	0.38	0.40	1.44	3.46	0.27	< .001
Living alone	0.20	− 0.11	0.85	1.51	0.25	0.13
Subjective visual impairment	− 0.07	− 0.54	0.28	− 0.64	0.21	0.53
Subjective hearing impairment	0.01	− 0.40	0.41	0.03	0.21	0.97
MDPQ-16 (points)	0.19	0.03	0.07	4.13	0.01	< .001
SCC (ref: none)	− 0.08	− 0.56	0.20	− 0.92	0.19	0.36

General linear model, Dependent variable; total score of financial management in PADA-D, adjusted *R*^2^ = 0.06, Overall model test; *F* = 5.33, *p* < .001

*CI* confidential interval, *LPM* low proficiency of mobile devices, *SCC* subjective cognitive complain, *MDPQ-16* short version of the mobile device proficiency questionnaire, *PADA-D* process analysis of daily activities for dementia, *SE* standardized error

### Detailed comparison of financial management ability

Table 3 compares the implementation rates of the 15 financial management actions in the PADA-D with four groups. No significant differences were found in the implementation rates by the groups for the processes “Handle cash”, “Use cash in daily life,” and “Understand household expenses.” In the “Use the bank and the post office” process, the percentage of participants who performed the action “Use the ATM for withdrawals/deposits/bank transfers/payments” was significantly lower in the LPM group (*p* < 0.05). Conversely, for the “Use electronic money” process, the robust and SCC groups performed better in all three actions.

**Table 3:** Comparison of money management skills in PADA-D with and without SCC or LPM

Process	Action	Overall, *n* = 529	Robust, *n* = 224	SCC, n = 173	LPM, *n* = 75	DI, *n* = 57	ES	95% CI	*p* value
Handle cash	(a) Take out the required number of coins	488 (92%)	216 (96%)	153 (88%)	68 (91%)	51 (89%)	0.14	0.08–0.22	0.019
	(b) Take out the required number of bills	504 (95%)	220 (98%)	161 (93%)	70 (93%)	53 (93%)	0.12	0.07–0.21	0.056
	(c) Get the correct amount of change	509 (96%)	216 (96%)	163 (94%)	74 (99%)	56 (98%)	0.09	0.04–0.17	0.281
Use cash on a daily life	(a) Mainly handle cash for purchasing of grocery	515 (97%)	218 (97%)	167 (97%)	73 (97%)	57 (100%)	0.06	0.05–0.14	0.623
	(b) Handle cash for rent and bill payments	344 (65%)	139 (62%)	113 (65%)	52 (69%)	40 (70%)	0.06	0.03–0.17	0.546
	(c) Handle cash for special events	494 (93%)	208 (93%)	165 (95%)	68 (91%)	53 (93%)	0.06	0.03–0.17	0.557
Understand household expenses	(a) Understand the necessary amount for living expenses	472 (89%)	206 (92%)	148 (86%)	69 (92%)	49 (86%)	0.10	0.05–0.20	0.142
	(b) Understand the amount of savings	496 (94%)	218 (97%)	159 (92%)	70 (93%)	49 (86%)	0.15	0.09–0.26	0.008
	(c) Understand assets	451 (85%)	203 (91%)	143 (83%)	62 (83%)	43 (75%)	0.14	0.08–0.24	0.013
Use the bank and the post office	(a) Understand where the seals/passbooks/cards have been stored	519 (98%)	222 (99%)	169 (98%)	73 (97%)	55 (96%)	0.07	0.04–0.18	0.478
	(b) Understand the procedures for transactions at the bank counter (for withdrawals, deposits, bank transfers/payment)	322 (61%)	133 (59%)	110 (64%)	42 (56%)	37 (65%)	0.06	0.03–0.17	0.600
	(c) Use the ATM for withdrawals/deposits/bank transfers/payments	494 (93%)	214 (96%)	168 (97%)	61 (81%)	51 (89%)	0.22	0.12–0.33	< 0.001*
		Adj. Res	1.71	**2.40**	** − 4.53**	− 1.26			
Use electronic money	(a) Tap card against the card reader	219 (41%)	103 (46%)	87 (50%)	21 (28%)	8 (14%)	0.24	0.18–0.32	< 0.001*
		Adj. Res	1.83	**2.89**	** − 2.54**	** − 4.44**			
	(b) Transfer some cash to the card	290 (55%)	138 (62%)	108 (62%)	29 (39%)	15 (26%)	0.26	0.19–0.34	< .001*
		Adj. Res	**2.69**	**2.45**	** − 3.03**	** − 4.58**			
	(c) Understand the balance on the card	272 (51%)	127 (57%)	97 (56%)	35 (47%)	13 (23%)	0.21	0.14–0.29	< 0.001*
		Adj. Res	**2.08**	1.49	− 0.89	** − 4.58**			

Numbers and percentages represent the number of people who reported having implemented actions

Statistical test performed Chi-square test of independence, ES used Cramer’s V, *p* values with * indicate statistically significant at ≤ 0.003 using the Bonfferoni correction

*Adj. Res.* adjusted residuals, *CI* confidence interval, *SCC* subjective cognitive complain, *LPM* low proficiency of mobile devices, *DI* dual impairment, *ES* effect size, *PADA-D* process analysis of daily activities for dementia

Bold text indicates *p* < 0.05

## Discussion

We investigated the relationship between financial management ability, mobile device proficiency, and SCC among community-dwelling older adults in this study. Contrary to our hypotheses, the LPM group had significantly lower financial management ability regardless of the SCC status. These results suggest that low mobile device proficiency is related to financial management ability and background factors such as gender and educational history. Moreover, the group that was proficient in using mobile devices had a significantly higher percentage of participants who used ATMs and electronic money among their financial management ability, regardless of SCC status. This finding suggests that proficiency with mobile devices helps people with SCC maintain a high level of financial management ability.

In this study, 43.2% of the participants had SCC, which was similar or slightly higher than that reported previously [6, 24, 25]. There are several reasons for this: First, the methods used to assess SCC varied across the studies. We used some of the measures used in previous cohort studies of Japanese populations. Thus, we cannot rule out the possibility that the results would differ if different measures were used. Second, background factors may affect the participants. Several previous reports have shown that women and older age are associated with higher memory complaints [8, 31–35]. Because 85% of the participants in this study were female, with a mean age of 72.4 years, it could be the reason for the higher proportion of SCC compared to that in the previous studies. Moreover, the proportion of people with sensory impairment was significantly higher among older adults with SCC. Because vision and hearing impairments are associated with subsequent cognitive impairment [36], early introduction of vision and hearing aids should be considered for patients with SCC.

Furthermore, the mean MDPQ-16 score was higher in this study population than that of previous studies [27]. This may be attributed to the rapid adoption of mobile devices among older adults. Previous studies have suggested that older adults have barriers to adopting and using digital technology [37]. However, many older adults are embracing digital technology in recent years to changing times [38]. This suggests that older adults are adapting to changing times. Nevertheless, our results showed that older adults (mean: 75.40 years) and those with fewer years of education (mean: 11.83 years) had lower proficiency of mobile devices. This may reflect the digital divide among older adults [39], and efforts to bridge the gap will be needed in the future. In particular, support in using mobile devices would be important for those aged 75 years and older and those with low levels of education to maintain high levels of money management ability.

Interestingly, the results suggest that participants’ ability to manage finances may be associated with the LPM rather than SCC. This suggests that older adults with SCC may partially compensate for managing money by using mobile devices. For example, when handling cash, those with SCC (without LPM) were less likely to handle coins as change. This may be because the electronic money eliminates the need to calculate balances and change. The use of ICT, such as mobile devices, to assist older adults with memory function can be effective [40, 41]. Mobile devices may be helpful for financial management in situations such as timely reminders, internet banking, and calculation, although this study did not explore them. Although shopping and transportation payments are being digitized to reduce costs, improve efficiency, and prevent COVID-19 infection, older adults with limited internet and smartphone access may be limited by their use of cashless payments [42]. Policymakers and rehabilitation staff need to provide financial management training for older adults less familiar with mobile devices so that they are not left behind in an increasingly digitalized living environment. However, when supporting older adults to manage their finances digitally, it is necessary to consider the importance of privacy and the fact that a certain percentage of the population does not have their own accounts [43]. Thus, assistance must be tailored considering the personal and environmental factors.

### Limitation

Our findings should be interpreted with several limitations. First, the cross-sectional design of this study makes it difficult to describe the causal effects of SCC and LPM on financial management. Second, most participants in this study were females. Although it has been reported that men are more financially literate than women, especially in Japan [44], our results showed that women have better financial management abilities than men. In Japan, 87% of CO-OP members are women, which may have biased the gender ratio despite the random selection. However, no differences in associated factors were found in analyses stratified by men and women. Third, older adults with SCCs are more likely to have psychosomatic stress [45], and including participants with psychiatric symptoms such as depression in this study may affect the results. Participants with depressive symptoms may go shopping less often and may have fewer opportunities for the demands of money management abilities. Future studies need to include brief screening for psychiatric symptoms. Finally, we did not include information on income level, which could affect mobile device ownership. It is possible that lower income groups have lower rates of mobile device ownership. Nevertheless, this study examined the relationship between older adults’ ability to manage finances and their proficiency with SCC and mobile devices providing useful suggestions to support the daily lives of older adults in today’s increasingly digital society.

## Conclusion

We used a self-administered questionnaire to examine the relationship between mobile device proficiency, SCC, and financial management ability among older adults. The results showed that older adults’ ability to manage finances was related to LPM rather than SCCs. Specifically, using ATMs and electronic money was limited, suggesting that older adults with LPM may need advanced financial management support. Policymakers and rehabilitation professionals need to consider ADL impairments not only by SCC status but also by the level of mobile device proficiency. Further studies are needed to consider gender and psychological assessment.

### Supplementary Information

Below is the link to the electronic supplementary material.

Supplementary file1 (DOCX 42 KB)

## Data Availability

The data that support the findings of this study are available on request from the corresponding author, S.S. The data are not publicly available due to restrictions on their containing information that could compromise the privacy of research participants.
