# Supplementary material for: Association of mobile device proficiency and subjective cognitive complaints with financial management ability among community-dwelling older adults: a population-based cross-sectional study
Source: Aging Clin Exp Res. 2024 Feb 17;36(1):44. doi: 10.1007/s40520-024-02697-8 (PMC10874308; doi:10.1007/s40520-024-02697-8)

# **Supplementary material**

Table S1

*Background of participants: comparison with and without SCC*

| Characteristic | non-SCC,  n = 299 | SCC,  n = 230 | *p*-value |
| --- | --- | --- | --- |
| Age | 71.94 (5.39) | 73.01 (6.00) | 0.06^a^ |
| Sex (Female) | 258 (86%) | 192 (83%) | 0.41^b^ |
| Education years | 12.76 (2.06) | 12.61 (2.16) | 0.38^a^ |
| Living alone | 46 (15%) | 51 (22%) | 0.74^b^ |
| Subjective Visual impairment | 180 (60%) | 176 (77%) | < .001^b^ |
| Subjective Hearing impairment | 86 (29%) | 108 (47%) | < .001^b^ |
| MDPQ-16 (score) | 20.80 (8.77) | 19.41 (8.18) | 0.10^a^ |
| Statistics presented: Mean (SD); n (%) | | | |
| Statistical tests performed: ^a^ Mann-Whitney U test; ^b^ chi-square test of independence  Abbreviations: SCC; subjective cognitive complain, MDPQ-16; short version of the Mobile Device Proficiency Questionnaire | | | |

Table S2

*Background of participants: comparison with and without LPM*

| Characteristic | non-LPM, N = 397^1^ | LPM, N = 132^1^ | p-value^2^ |
| --- | --- | --- | --- |
| Age | 71.41 (5.15) | 75.40 (6.17) | < .001^a^ |
| Sex (Female) | 337 (85%) | 113 (86%) | 0.95^b^ |
| Education years | 12.99 (1.98) | 11.83 (2.21) | < .001^a^ |
| Living alone | 66 (17%) | 31 (23%) | 0.10^b^ |
| Subjective Visual impairment | 271 (68%) | 85 (64%) | 0.48^b^ |
| Subjective Hearing impairment | 140 (35%) | 54 (41%) | 0.29^b^ |
| SCC (+) | 173 (44%) | 57 (43%) | 0.94^b^ |
| Statistics presented: Mean (SD); n (%) | | | |
| Statistical tests performed: ^a^ Mann-Whitney U test; ^b^ chi-square test of independence  Abbreviations: LPM; low proficiency of mobile devices, SCC; subjective cognitive complaints | | | |

Table S3

*Percentage of mobile device applications currently used*

|  | Overall, n = 529 | Robust, n = 224 | SCC, n = 173 | LPM, n = 75 | DI, n = 57 |
| --- | --- | --- | --- | --- | --- |
| 1. Messages/Texting (e.g., Facebook Messenger, WhatsApp, Line) | 418 (79%) | 207 (92%) | 156 (90%) | 32 (43%) | 23 (40%) |
| 1. E-mail (e.g., Gmail, Outlook) | 371 (70%) | 180 (80%) | 126 (73%) | 39 (52%) | 26 (46%) |
| 1. Phone calls | 256 (48%) | 142 (63%) | 91 (53%) | 18 (24%) | 5 (8.8%) |
| 1. Internet browsers (e.g., Safari, Chrome) | 169 (32%) | 94 (42%) | 66 (38%) | 5 (6.7%) | 4 (7.0%) |
| 1. Cameras | 439 (83%) | 209 (93%) | 159 (92%) | 43 (57%) | 28 (49%) |
| 1. Social networking services (e.g., Facebook, Twitter, Instagram) | 108 (20%) | 56 (25%) | 46 (27%) | 3 (4.0%) | 3 (5.3%) |
| 1. Video viewing (e.g., YouTube, Netflix) | 174 (33%) | 96 (43%) | 70 (40%) | 2 (2.7%) | 6 (11%) |
| 1. Online games | 47 (8.9%) | 24 (11%) | 22 (13%) | 1 (1.3%) | 0 (0%) |
| 1. News and Weather | 409 (77%) | 212 (95%) | 156 (90%) | 25 (33%) | 16 (28%) |
| 1. Maps/Navigation | 301 (57%) | 163 (73%) | 118 (68%) | 10 (13%) | 10 (18%) |
| 1. Online shopping (e.g., Amazon, Rakuten) | 128 (24%) | 77 (34%) | 51 (29%) | 0 (0%) | 0 (0%) |
| 1. Financial transactions | 30 (5.7%) | 16 (7.1%) | 14 (8.1%) | 0 (0%) | 0 (0%) |
| 1. Contents (e-books, music) | 45 (8.5%) | 23 (10%) | 20 (12%) | 0 (0%) | 2 (3.5%) |
| 1. Auctions | 23 (4.3%) | 13 (5.8%) | 10 (5.8%) | 0 (0%) | 0 (0%) |
| 1. Electronic government communication (e.g., tax payment, address change) | 60 (11%) | 37 (17%) | 22 (13%) | 0 (0%) | 1 (1.8%) |
| Statistics presented: n (%), SCC; subjective cognitive complain, LPM; low proficiency of mobile devices, DI; dual impairment | | | | | |

Table S4

*Factors associated with the ability to manage finances: stratified by sex*

|  | β | 95% CI | | t | SE | *p*-value |
| --- | --- | --- | --- | --- | --- | --- |
|  |  | lower | upper |  |  |  |
| Model 1: Female |  |  |  |  |  |  |
| Age | -0.02 | -0.12 | 0.08 | -0.36 | 0.02 | 0.72 |
| Education years | 0.10 | 0.01 | 0.20 | 2.18 | 0.05 | 0.03 |
| Living alone | 0.13 | -0.11 | 0.37 | 1.03 | 0.26 | 0.30 |
| Subjective visual impairment | 0.03 | -0.17 | 0.24 | 0.33 | 0.22 | 0.75 |
| Subjective hearing impairment | 0.05 | -0.15 | 0.25 | 0.51 | 0.21 | 0.61 |
| MDPQ-16 (points) | 0.15 | 0.05 | 0.25 | 2.94 | 0.01 | < .01 |
| SCC (ref: none) | -0.12 | -0.31 | 0.07 | -1.21 | 0.20 | 0.23 |
| Model 2: Male |  |  |  |  |  |  |
| Age | 0.01 | -0.09 | 0.10 | 0.13 | 0.02 | 0.90 |
| Education years | 0.10 | 0.01 | 0.18 | 2.16 | 0.05 | 0.03 |
| Living alone | 0.17 | -0.05 | 0.39 | 1.50 | 0.25 | 0.13 |
| Subjective visual impairment | -0.04 | -0.23 | 0.14 | -0.46 | 0.21 | 0.65 |
| Subjective hearing impairment | 0.03 | -0.16 | 0.21 | 0.27 | 0.21 | 0.79 |
| MDPQ-16 (points) | 0.18 | 0.09 | 0.28 | 3.93 | 0.01 | < .001 |
| SCC (ref: none) | -0.10 | -0.27 | 0.07 | -1.14 | 0.20 | 0.25 |
| General linear model, Dependent variable; total score of financial management in PADA-D, Model 1: adjusted R^2^ = 0.04, Overall model test; F = 3.32, p = 0.002, Model 2: adjusted R^2^ = 0.06, Overall model test; F = 4.75, p < .001  Abbreviations: CI; confidential interval, LPM; low proficiency of mobile devices, SCC; subjective cognitive complain, MDPQ-16; Short Version of the Mobile Device Proficiency Questionnaire, PADA-D; Process Analysis of Daily Activities for Dementia, SE; standardized error | | | | | | |

# Figure S1

*Distribution of MDPQ-16 scores in the participants <65 years old*


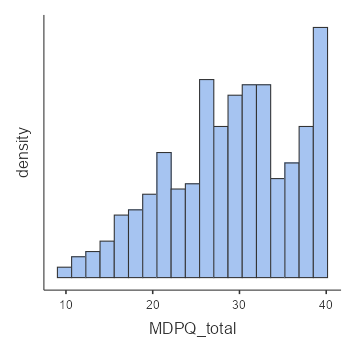


# Figure S2

*Distribution of MDPQ-16 scores in the participants ≥65 years old*


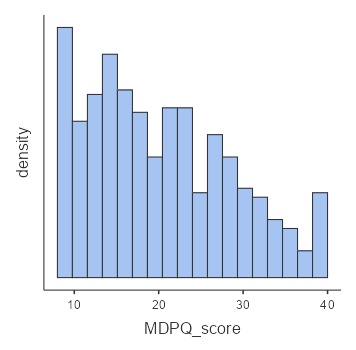

Supplement: Supplementary file 1 — Supplementary file1 (DOCX 42 KB) [file 40520_2024_2697_MOESM1_ESM.docx]
